# Editorial: Visualizing big culture and history data

**DOI:** 10.3389/fdata.2025.1563730

**Published:** 2025-02-04

**Authors:** Florian Windhager, Steffen Koch, Sander Münster, Eva Mayr

**Affiliations:** ^1^Center for Cultures and Technologies of Collecting, Department for Arts and Cultural Studies, University for Continuing Education, Krems, Austria; ^2^Visualization Research Center (VISUS), University of Stuttgart, Stuttgart, Germany; ^3^Junior Professorship for Digital Humanities, University of Jena, Jena, Germany

**Keywords:** visualization, culture, history, digital humanities, big data

In recent years, the visualization of cultural and historical data has become a vibrant and innovative field of research. The expanding efforts of digitization by archives, museums, libraries, and other repositories of human knowledge have not only made vast amounts of cultural-historical data accessible but also demand scalable methods of analysis and representation. In this context, visualization emerges as a critical tool—for analysis, but also for generating new questions, fostering interdisciplinary collaboration, and engaging diverse audiences.

## 1 Rethinking visualization for cultural and historical data

Visualizing big, multi-sourced data of the past is not simply a technical challenge, it is an intellectual and interpretive one. While early efforts in digital humanities focused on digitizing and representing discrete collections, the growing complexity of the field manifests in projects going beyond individual datasets. Nowadays, scholars connect local case studies, create contextual linkages, and connect microhistorical details to macrohistorical frameworks, as seen in large-scale initiatives such as the European Data Space for Cultural Heritage and Europeana,^1^ the European Collaborative Cloud for Cultural Heritage,^2^ or the Time Machine Organization.^3^ These efforts herald what Kaplan and Di Lenardo (2020) refer to as a “4D mirror world,” a big data framework of the past, that maps the interconnected fabric of history through time and space.

Addressing related challenges is urgent for several reasons. First, the sheer scale, complexity, and richness of cultural data aggregations—from digitized manuscripts to gigapixel artworks—demand tools to navigate, explore, and interpret them effectively. Second, the datasets' multi-layered diversity—including textual, visual, relational, and geographical components—calls for interdisciplinary approaches integrating methods from the humanities, data science, and visualization. Third, historical data is often incomplete, ambiguous, or contested, reflecting the humanities' discursive and interpretive nature. Finally, visualization as a medium itself poses important questions: What does it mean to represent and interpret history visually? How do we balance clarity with complexity, precision with uncertainty, and the local with the global?

The visualization of cultural and historical data also raises ethical questions. Visualizations are not neutral; they reflect the decisions, biases, and goals of both their creators and the initial data collectors. They foreground certain perspectives and narratives, obscuring others, and play a major role in shaping how audiences (scholars, students, and the public) engage with the past. This makes visualization design not just a technical endeavor but also a deeply critical and reflective one.

This Research Topic explores these intersections of technology, theory, and interpretation. It showcases pioneering works combining humanities scholarship with computational methods and visualization to uncover patterns, connections, and narratives within complex cultural datasets. Together, they offer insights into how the field can reshape the ways we study, share, and understand cultural and historical knowledge.

## 2 Key themes explored in this Research Topic

Several recurring themes emerge across the contributions in this Research Topic, reflecting both opportunities and challenges of visualizing big culture and history data. One is the *integration of diverse datasets and perspectives* by linking localized or fragmented datasets into larger knowledge graphs. *impresso* by Düring et al. enriches text reuse data semantically to explore historical newspapers. The LuxTIME project by Aurich and Horaniet Ibañez brings together a range of archival sources to study the exposome during industrialization. These projects underscore the importance of integrating multiple sources to address complex historical questions.

*Interdisciplinary collaboration* is another recurring motif. LuxTIME highlights the potential of data visualization to bridge diverse fields–from public health to environmental history (Aurich and Horaniet Ibañez). Schwandt and Wachter discuss how visualizations serve as tools for “productive irritation” in collaborative DH projects, which challenge traditional notions of clarity and objectivity by embracing ambiguity and provoking deeper engagement with the data.

Another recurring challenge is the combination of *overview and detail*. *impresso* (Düring et al.) illustrates how combining close and distant reading techniques can reveal both granular details and broader patterns of intertextuality in historical media. Benito-Santos et al. bridge computational aesthetics with traditional close reading practices of poems, offering insights into the interplay of structure and creativity in individual poems, but also across a larger corpus. Similarly, Chau et al. exemplify how panoramic visualization strategies, rooted in 19th-century traditions, can be remediated using gigapixel imaging and machine learning to allow for both contemplation of the whole picture and immersion into striking details.

Finally, a recurring theme is *uncertainty*: Schwandt and Wachter argue that visualizations provoke uncertainty not only about datasets but also about their own interpretative framework. The visualization of uncertain data is also discussed by Benito-Santos et al. and by Aurich and Horaniet Ibañez.

Despite these shared themes, the contributions also highlight the field's diversity: From scalability and automation to immersion and user experience, from theoretical reflections to visualization design studies. This diversity reflects the field's potential to address a wide range of research questions. It also highlights the importance of tailoring visualizations to specific datasets, audiences, and interpretive goals.

## 3 Toward a bigger picture of cultural and historical data

This Research Topic invites readers to envision a future where the visual representation of and reasoning with big cultural and historical data are integral to scholarly practice. The featured articles demonstrate the potential of these practices to uncover connections, generate hypotheses, and engage audiences in new ways. They also highlight the importance of theoretical reflection, reminding us that visualizations are not neutral arbiters but active participants in knowledge production. As we look to the future, the field faces several challenges:

- Data Quality: Historical datasets often suffer from gaps, inconsistencies, and biases (see also Münster et al., 2024). Visualizations must acknowledge and explicate these uncertainties, rather than obscuring them.
- Standardization and Interoperability: Different historical sources use different data standards and models. Researchers have to link datasets across institutions and disciplines and effectively connect multiple visualization techniques (Windhager et al., 2024).
- Balancing Scale and Detail: The need to accommodate both macroscopic trends and microscopic details poses a fundamental design challenge. Successful visualizations must enable seamless transitions between distant and close reading, facilitating both broad insights and granular analysis (Bludau et al., 2020).
- Interdisciplinary Collaboration: Linking and visualizing data requires technical expertise beyond the reach of many humanities scholars, thereby making collaborations between data scientists, historians, and designers essential (McCarty, 2016).
- Ethical Considerations: Visualizations shape interpretation. Scholars must remain vigilant about the ethical implications of their choices, ensuring that their work represents historical subjects with nuance and respect, while also embracing and disclosing aspects of interpretive agency (Drucker, 2020).

As cultural historians increasingly operate within interconnected historical and cultural datasets, visualization plays a critical role in shaping how we understand and interpret them. By navigating the opportunities and challenges of this transformative domain, we can create a dynamic, multimodal discourse that bridges technology, culture, and history. This Research Topic aims to deepen this conversation, offering fresh approaches and valuable insights for researchers and practitioners alike.

## Footnotes

^1^ https://www.europeana.eu/

^2^ https://www.echoes-eccch.eu/

^3^ https://www.timemachine.eu/
